# Supplementary material for: Measuring Quality of Life: Incorporating Objectively Measurable Parameters within the Cross-Sectional Bern Cohort Study 2014 (BeCS-14)
Source: Int J Environ Res Public Health. 2024 Jan 15;21(1):94. doi: 10.3390/ijerph21010094 (PMC10815394; doi:10.3390/ijerph21010094)
Supplement: Supplementary file 1 [file ijerph-21-00094-s001.zip › Table S1.docx]

**Table S_1:** Overview of BFS assessment items sorted by subdomains of BFS

| **Nr.** | **BFS subdomain** | **Item** | **Units** | **Method/ Test procedure** |
| --- | --- | --- | --- | --- |
| 01 | I | Systolic blood pressure | mmHg | Blood pressure measurement |
| 02 | I | Diastolic blood pressure | mmHg |  |
| 03 | I | Puls Performance Index (PPI) | Δp/performance time | Knee bend test |
| 04 | I | Pulse rate difference (Δp) | n/min |  |
| 05 | I | Resting heart rate | bpm |  |
| 06 | I | Performance time | Sec |  |
| 07 | I | Vital capacity | l | Spirometry |
| 08 | I | Hand grip strength(sum left+right) | kp | Dynamometry |
| 09 | I | Fat mass | kg | Bioimpedance measurement |
| 10 | I | Body cell mass (active cell mass) | kg |  |
| 11 | I | Decayed Missing Filled Teeth | n | DMF determination |
| 12 | II | Vision rechts | % | Visual acuity determination |
| 13 | II | Vision links | % |  |
| 14 | II | Hörverlust rechts 2048 Hz | % | Audiometry |
| 15 | II | Hörverlust rechts 4096 Hz | % |  |
| 16 | II | Hörverlust links 2048 Hz | % |  |
| 17 | II | Hörverlust links 4096 Hz | % |  |
| 18 | II | Start rate | Hz | Tapping-Test |
| 19 | II | Test motivation | Hz |  |
| 20 | II | Tapping – basic rate, part 3 | Hz |  |
| 21 | II | Viseomotor coordination ability (time) [sec] | Sec | Viseomotor coordination test according to Pögelt and Roth |
| 22 | II | Viseomotor coordination ability (mistakes) [n] | Sec |  |
| 23 | III | Optical reaction time | mSec | Determination of reaction times according to Pögelt and Roth |
| 24 | III | Acoustical reaction time | mSec |  |
| 25 | III | Pursuing reaction time | mSec |  |
| 26 | III | Verbal reaction time | Sec | colour-word test according to Stroop (modified) |
| 27 | III | Cognitive reaction time | Sec |  |
| 28 | III | Cognitive switching capability | Sec |  |
| 29 | III | Ability to concentrate (time) | Sec | Landolt concentration-time test (modified) |
| 30 | III | Ability to concentrate (mistakes) | n |  |
| 31 | III | Strategic thinking | Sec | stepping-stone-maze (labyrinth test) according to Pögelt and Roth |
| 32 | III | Memory performance | n |  |
| 33 | III | Orientation capability | n |  |
| 34 | III | Change over capability | Sec |  |
| 35 | IV | Physical wellbeing | [score] | Complaint questionnaire according to Höck and Hess (Beschwerdefragebogen, BFB) |
| 36 | IV | Emotional wellbeing | [score] |  |
| 37 | IV | Sense of coherence | [score] | SOCL9 |
| 38 | IV | Stress disposition [score] | [score] | Giessen-Test |
| 39 | IV | Stress exposition (social stress/ resonance) | [score] |  |
| 40 | IV | Social dominance | [score] |  |
| 41 | IV | Social power | [score] |  |
| 42 | IV | Social activity / duties | [score] | Leningrad coverage scale (Leningrader Erfassungsskala) |
| 43 | IV | Social activity / leisure | [score] |  |

BFB = compliant questionnaire DMF = decayed missed filled, Hz = Hertz, kp = Kilopascal, kg = Kilogramm, mmHg = Milimeter mercury ml = Milliliter, n = number, (m)sek = (milli-)seconds, SOCL9 = Sense of Coherence Scale – Leipziger Kurzform, I: physical parameters, II: sensory physiology parameter III: cognitive and mental parameters, IV: emotional-social parameters
