# Supplementary material for: Measuring Quality of Life: Incorporating Objectively Measurable Parameters within the Cross-Sectional Bern Cohort Study 2014 (BeCS-14)
Source: Int J Environ Res Public Health. 2024 Jan 15;21(1):94. doi: 10.3390/ijerph21010094 (PMC10815394; doi:10.3390/ijerph21010094)
Supplement: Supplementary file 1 [file ijerph-21-00094-s001.zip › Table S2.docx]

**Table_S_2: Presentation of individual proceedings of BFS**

Blood pressure measurement

Measured variables: 01 - systolic blood pressure [mmHg].

02 - diastolic blood pressure [mmHg]

Devices: classic blood pressure monitor, stethoscope

Procedure: The examiner measures the systolic and diastolic blood pressure of the subject according to systolic and diastolic blood pressure according to Riva-Rocci.

Documentation: Input of the values into the diagnostic software VITAL.EXPERTISE (= V.E.)

Knee bend test

Measured variables: 03 - Pulse Performance Index [n] (quotient of pulse frequency difference and load time)

04 - Pulse rate difference [bpm]

05 – Resting heart rate [bpm]

06 – Performance time [sec]

Devices: stopwatch, hourglass (15 sec)

Procedure: The subject performs 20 squats. The speed and intensity are to be individually adapted to the goal of submaximal stimulation of the heart rate. Subsequently, the examiner measures the pulse of the test person manually on his wrist using the hourglass.

Documentation: V.E.

Spirometry

Measured variables: 07 - Vital capacity [l/%]

Devices: Spirometer, nasal clamp

Procedure: After maximum inspiration the subject performs a maximum expiration into the spirometer while standing. Out of three trials the best one counts.

Documentation: V.E.

Dynamometry

Measured variables: 08 - Hand grip strength [kp].

Devices: Hand force dynamometer

Procedure: The test person holds the dynamometer clamped between finger and palm and squeezes it in a standing position with bent arm with maximum force. The best attempt out of three counts.

Documentation: V.E.

Bioimpedance measurement

Measured variables: 09 - Fat mass [kg]

10 - Active cell mass [kg]

Devices: Bioimpedance meter, electrodes

Procedure: Automatic measurement with the subject lying down.

Documentation: V.E.

DMF determination

Measured variables: 11 - Decayed Missing Filled Teeth (DMF [n]).

Instruments: spatula, mirror (if required)

Procedure: The examiner determines the number of decayed (D), missing (M) and filled (F) teeth in cooperation with the patient.

Documentation: V.E.

Visual acuity determination

Measured variables: 12 - Visual acuity right [%]

13 - Visual acuity left [%]

Devices: Visual acuity chart (letters)

Procedure: Determination of visual acuity using a conventional

visual acuity chart. Visual acuity determination is not required

for subjects with congenital eye diseases or eye diseases that

developed before the age of 35.

Documentation: V.E.

Audiometry

Measured variables: 14 - Hearing loss right 2048 Hz [%]

15 - Hearing loss right 4096 Hz [%]

16 - Hearing loss left 2048 Hz [%]

17 - Hearing loss left 4096 Hz [%]

Equipment: audiometer, headphones, audiogram form

Procedure: The examiner determines the intensity threshold (dB) of the tones at the frequencies 512, 1024, 2048 and 4096 Hz. The subject listens to the tones via air conduction headphones first in the right, then in the left ear.

Documentation: V.E.

Tapping

Measured variables: 18 - Start rate [Hz]

19 - Test motivation [Hz]

20 - Psychomotor endurance/ Tapping basic rate [Hz]

Devices: Tapping pen (electronic recording)

Procedure: The test person taps the tapping mat with the tapping instrument for one minute at maximum frequency.

Documentation: The values are stored automatically. (= automatically stored)

Viseomotor coordination test according to Pögelt and Roth

Measured variables: 21 - Viseomotor coordination / time [sec]

22 - Viseomotor coordination / mistakes [n]

Devices: Touchpad, pen (electronic recording)

Procedure: The respondent moves the pen along the test line in as short a time as possible with as few errors as possible (= deviation from the line to the side or upwards) along the testline. A signal sounds for each error.

Documentation: automatically stored

Determination of reaction times according to Pögelt and Roth

Measured variables: 23 - Optical reaction time [msec]

24 - Acoustic reaction time [msec]

25 - Pursuing reaction time [msec]

Devices: Computer, program with reaction tasks

Procedure: The subject is shown a large red dot on the

computer screen 10 times at different time intervals (optical

reaction) or is played a sound by the computer 10 times at

different time intervals (acoustic reaction). In response to these

stimuli, the subject presses the Space-button as quickly as

possible. For optical follow-up reaction, the subject has to stop

a rotating pointer as precisely as possible after performing

three complete rotations at "12 o'clock".

Documentation: automatically stored

Color-word test according to Stroop (modified)

Measured variables: 26 - Verbal reaction time [sec]

27 - Cognitive reaction time [sec].

28 - Cognitive switching capability [sec].

Instruments: Test chart according to Stroop (modified):

- 3 lines of 10 words each (RED, GREEN, BLUE) in black printing ink

- 3 lines of 10 boxes each in red, green or blue ink

- 3 lines of 10 words each (RED, GREEN, BLUE) in red, green or blue ink

Stopwatch

Procedure: The respondent reads out the words as quickly as

possible (verbal reaction ability) or names the colors of the color

blocks (cognitive reaction ability) or names the printing color of the

words (cognitive conversion ability).

Documentation: V.E.

Landolt Concentration Time Test (modified)

Measured variable: 29 – Ability to concentrate / time [sec].

30 – Ability to concentrate / mistakes [n]

Instruments: Landolt test sheet: A4 sheet with 600 Landolt rings

(circular ring with opening to the right, left, bottom or top) in 30 lines

of 20 rings each, 20 rings are open to the top (12 o'clock), stopwatch.

Procedure: The subject ticks off as quickly as possible all Landolt rings that have their opening at 12 o'clock.

Documentation: V.E.

Stepping-stone-maze (labyrinth test) according to Pögelt and Roth

Measured variables: 31 – Strategic thinking [sec].

32 - Memory performance

33 - Orientation capability

34 – Change over capability

Devices: Touchpad, pen (electronic recording)

Procedure: The respondent searches for the way from the starting point (bottom left) to the target point (top right) in as short a time as possible and with as few repetition errors as possible. Wrong waypoints are signaled by muffled tones, correct waypoints by bright tones.

Documentation: automatically stored

Complaints Questionnaire (BFB) according to Höck and Hess

Measured variables: 35 - Physical wellbeing (the higher, the more complaints)

36 - Emotional wellbeing (the higher, the more complaints)

Instruments: standardized questionnaire according to Höck and Hess, Evaluation template

Procedure: The respondent fills out the questionnaire and the examiner evaluates it.

Documentation: V.E.

Sense of Coherence Scale - Leipzig Short Form (SOCL9)

Measured variables: 37 – Sense of coherence

Instruments: SOCL9 questionnaire, evaluation template

Procedure: The respondent fills out the questionnaire and the examiner evaluates it.

Documentation: V.E.

Giessen Test

Measures: 38 - Stress disposition (Self-control)

39 - Social exposition (social stress/resonance)

40 - Social dominance

41 - Social power

Instruments: Giessen test, evaluation template

Procedure: The respondent fills out the questionnaire and the examiner evaluates it.

Documentation: V.E.

Leningrad Coverage Scale

Measures: 42 - Social activity - duties

43 - Social activity - leisure

Instruments: Computerized questionnaire

Procedure: The respondent fills in the questionnaire on the computer.

Documentation: automatically stored
