# Supplementary material for: Measuring Quality of Life: Incorporating Objectively Measurable Parameters within the Cross-Sectional Bern Cohort Study 2014 (BeCS-14)
Source: Int J Environ Res Public Health. 2024 Jan 15;21(1):94. doi: 10.3390/ijerph21010094 (PMC10815394; doi:10.3390/ijerph21010094)
Supplement: Supplementary file 1 [file ijerph-21-00094-s001.zip › Table S3.docx]

**Table_S_3 Single items of the bio-functional status (BFS) and calculated bio-functional age (BFA)**

|  | Total (N = 630) | | | | | Subgroup 1 (N = 447) | | | | Subgroup 2 (N = 227) | | | |
| --- | --- | --- | --- | --- | --- | --- | --- | --- | --- | --- | --- | --- | --- |
| Bio-functional status (BFS) item | N | Mean | SD | 5th-95th  percentile | Min - Max | N | Mean | SD | Min-Max | N | Mean | SD | Min-Max |
| **Physical parameters** | | | | | | | | | | | | | |
| Systolic blood pressure [mmHg] | 619 | 118.9 | 13.8 | 99.0 - 144.0 | 82 - 169 | 447 | 119.6 | 14.0 | 82 - 169 | 227 | 123.3 | 15.3 | 82 - 169 |
| Diastolic blood pressure [mmHg] | 619 | 74.4 | 10.5 | 59.0 - 93.0 | 47 - 107 | 447 | 74.7 | 10.7 | 48 - 107 | 227 | 77.8 | 11.5 | 48 - 107 |
| Resting heart rate (p0) [n/min] | 614 | 68.4 | 10.4 | 52.8 - 86.0 | 42 - 107 | 446 | 68.5 | 10.6 | 45 – 107 | 226 | 69.6 | 10.3 | 45 - 102 |
| Pulse rate difference (Δp) | 607 | 121.0 | 18.5 | 92.0 - 152.0 | 68.0 – 204.0 | 441 | 121.9 | 18.3 | 68 - 188 | 223 | 118.6 | 17.4 | 68 - 172 |
| Performance time [sec] | 607 | 24.1 | 6.0 | 16.6 –- 34.3 | 12.1 – 63.0 | 441 | 24.2 | 6.1 | 12.1 – 57.0 | 223 | 26.5 | 6.8 | 12.1 – 57.0 |
| Pulse performance index  (PPI) [Δp/performance time] | 607 | 2.33 | 0.97 | 1.00 - 4.09 | 0.1 – 8.3 | 441 | 2.34 | 0.95 | 0.1 – 6.5 | 223 | 1.98 | 0.85 | 0.1 – 5.4 |
| Vital capacity [l] | 598 | 4.20 | 1.19 | 2.50 – 6.70 | 1.7-7.5 | 439 | 4.20 | 1.17 | 1.7 – 7.5 | 223 | 3.85 | 0.94 | 2.1 – 6.8 |
| Hand grip strength, both sides [KP] | 607 | 71.5 | 35.2 | 22.0 – 139.2 | 2.0 – 140.0 | 436 | 73.9 | 35.1 | 3.0 – 140.0 | 220 | 66.8 | 31.8 | 3.0 – 140.0 |
| Body cell mass (%) | 594 | 40.7 | 4.6 | 32.8 – 48.0 | 27.8 – 55.0 | 431 | 40.7 | 4.7 | 27.8 – 52.9 | 219 | 39.7 | 4.9 | 27.8 – 52.9 |
| Body cell mass – active cell mass [kg] | 594 | 27.7 | 5.0 | 22.0 – 37.7 | 19.5 – 48.5 | 431 | 27.9 | 5.0 | 19.5 – 48.5 | 219 | 27.7 | 4.5 | 19.5 – 41.6 |
| Body cell mass – body water [kg] | 594 | 35.1 | 6.4 | 27.9 – 47.8 | 24.8 – 61.8 | 431 | 35.4 | 6.4 | 24.8 – 61.8 | 219 | 35.1 | 5.8 | 24.8 – 42.5 |
| Body cell mass – lean body mass [kg] | 594 | 48.0 | 8.7 | 38.2 – 65.4 | 33.8 – 84.5 | 431 | 48.4 | 8.7 | 33.8 – 84.5 | 219 | 48.0 | 7.9 | 33.8 – 71.8 |
| Fat mass (%) | 594 | 29.4 | 8.0 | 16.4 – 43.0 | 4.8 – 51.4 | 431 | 29.4 | 8.2 | 8.5 – 51.4 | 219 | 31.2 | 8.4 | 8.5 – 51.4 |
| Fat mass [kg] | 594 | 20.5 | 8.4 | 10.3 – 36.4 | 1.9 – 56.5 | 431 | 20.8 | 8.5 | 4.8 – 56.5 | 219 | 22.5 | 9.1 | 5.9 – 56.5 |
| BIA RC 50 (Ohm) | 594 | 608.5 | 86.0 | 465.8 – 744.5 | 368 – 869 | 431 | 603.1 | 83.6 | 368 - 840 | 219 | 596.7 | 79.3 | 404 - 817 |
| BIA XC 50 (Ohm) | 594 | 63.1 | 10.8 | 47.0 – 80.0 | 30 – 116 | 431 | 62.3 | 10.8 | 30 - 116 | 219 | 58.4 | 10.3 | 30 - 116 |
| Teeth status - decayed, missing or  filled teeth (n) | 624 | 10.0 | 8.1 | 0.0 – 28.0 | 0 – 32 | 447 | 9.9 | 8.0 | 0 - 32 | 227 | 14.5 | 7.7 | 1 - 32 |
| Body weight (kg) | 625 | 68.4 | 13.2 | 51.0 – 94.0 | 36.0 – 120.0 | 447 | 69.0 | 13.0 | 36.0 – 113.0 | 227 | 70.0 | 12.9 | 36.0 – 110.0 |
| Body height (cm) | 625 | 169.8 | 8.6 | 157.0 – 185.0 | 150 – 195 | 447 | 170.0 | 8.7 | 150 - 195 | 227 | 168.1 | 8.0 | 152 - 195 |
| Body mass index [kg/m2] | 625 | 23.7 | 4.1 | 18.7 – 31.2 | 12.3 – 47.0 | 447 | 23.9 | 4.1 | 12.3 – 47.0 | 227 | 24.8 | 4.4 | 12.3 – 47.0 |
| **Sensory physiology and psychomotor parameters** | | | | | | | | | | | | | |
| Vision left [%] | 374 | 81.0 | 30.2 | 10.0 – 100.0 | 0.0 – 100.0 | 262 | 83.2 | 28.9 | 0.0 – 100.0 | 150 | 75.0 | 32.4 | 0.0 – 100.0 |
| Vision right [%] | 373 | 80.9 | 29.3 | 12.0 – 100.0 | 0.0 – 100.0 | 261 | 84.5 | 26.3 | 0.0 – 100.0 | 149 | 76.5 | 29.7 | 0.0 – 100.0 |
| Hearing acuity -HV1024 left (Db) | 558 | 16.0 | 8.7 | 5.0 – 30.5 | 0.0 – 100.0 | 436 | 15.7 | 8.9 | 0.0 -100.0 | 227 | 18.2 | 10.6 | 0.0 – 100.0 |
| Hearing acuity -HV1024 left (%) | 558 | 1.65 | 2.54 | 0.00 – 5.40 | 0.0 – 30.0 | 436 | 1.60 | 2.67 | 0.0 – 30.0 | 227 | 2.26 | 3.38 | 0.0 – 30.0 |
| Hearing acuity -HV1024 right (Db) | 558 | 17.5 | 8.6 | 7.0 – 30.0 | 0.0 – 77.0 | 436 | 17.2 | 8.8 | 0.0 – 77.0 | 226 | 19.4 | 9.7 | 0.0 – 77.0 |
| Hearing acuity -HV1024 right (%) | 558 | 1.97 | 2.77 | 0.00 – 5.40 | 0.0 – 27.2 | 436 | 1.91 | 2.90 | 0.0 – 27.2 | 226 | 2.52 | 3.34 | 0.0 – 27.2 |
| Hearing acuity -HV2048 left (Db) | 555 | 17.0 | 9.9 | 5.0 – 36.0 | 0.0 – 100.0 | 433 | 16.8 | 10.0 | 0.0 – 100.0 | 224 | 20.7 | 11.4 | 0.0 – 100.0 |
| Hearing acuity - HV2048 left [%] | 555 | 2.77 | 4.01 | 0.20 – 9.80 | 0.0 – 40.0 | 433 | 2.71 | 4.05 | 0.0 – 40.0 | 224 | 4.06 | 5.04 | 0.0 – 40.0 |
| Hearing acuity - HV2048 right (Db) | 556 | 18.3 | 10.0 | 6.0 – 37.2 | 0.0 – 88.0 | 434 | 18.0 | 10.3 | 0.0 – 88.0 | 224 | 21.7 | 11.6 | 0.0 – 88.0 |
| Hearing loss right 2048 Hz [%] | 556 | 3.19 | 4.45 | 0.20 – 10.26 | 0.0 – 39.2 | 434 | 3.14 | 4.62 | 0.0 – 39.2 | 224 | 4.59 | 5.48 | 0.0 – 39.2 |
| Hearing acuity -HV4096 left (Db) | 555 | 17.9 | 13.5 | 3.0 – 44.0 | 0.0 – 100.0 | 433 | 17.6 | 13.7 | 0.0 – 100.0 | 224 | 24.5 | 14.2 | 0.0 – 100.0 |
| Hearing loss left 4096 Hz [%] | 555 | 1.35 | 2.33 | 0.05 – 6.40 | 0.0 – 15.0 | 433 | 1.32 | 2.30 | 0.0 – 15.0 | 224 | 2.21 | 2.66 | 0.0 – 15.0 |
| Hearing acuity -HV4096 right (Db) | 556 | 18.4 | 13.4 | 3.0 – 45.2 | 0.0 – 100.0 | 434 | 18.1 | 13.5 | 0.0 – 100.0 | 224 | 23.9 | 13.5 | 0.0 – 90.0 |
| Hearing loss right 4096 Hz [%] | 556 | 1.35 | 2.40 | 0.05 – 6.40 | 0.0 – 15.0 | 434 | 1.32 | 2.38 | 0.0 – 15.0 | 224 | 2,10 | 2.72 | 0.0 – 14.9 |
| Hearing acuity -HV512 left (Db) | 558 | 14.9 | 7.5 | 5.0 – 26.0 | 0.0 – 100.0 | 436 | 14.7 | 7.8 | 0.0 – 100.0 | 227 | 16,3 | 9.3 | 0.0 - 100.0 |
| Hearing acuity -HV512 left (%) | 558 | 1.85 | 2.66 | 0.20 – 4.90 | 0.0 – 40.0 | 436 | 1.84 | 2.87 | 0.0 – 40.0 | 227 | 2.35 | 3.66 | 0.0 – 40.0 |
| Hearing acuity -HV512 right (Db) | 558 | 18.1 | 7.7 | 8.0 – 30.0 | 0.0 – 79.0 | 436 | 18.0 | 7.9 | 0.0 – 79.0 | 226 | 19.8 | 8.4 | 0.0 – 79.0 |
| Hearing acuity -HV512 right (%) | 558 | 2.81 | 3.03 | 0.40 – 7.20 | 0.0 – 35.8 | 436 | 2.80 | 3.17 | 0.0 – 35.8 | 226 | 3.42 | 3.37 | 0.0 – 35.8 |
| Start rate [Hz] | 615 | 6.6 | 0.9 | 5.2 – 8.1 | 2.0 – 9.2 | 445 | 6.7 | 0.9 | 2.4 – 9.2 | 225 | 6.5 | 0.8 | 2.4 – 9.0 |
| Test motivation [Hz] | 615 | 5.9 | 0.7 | 4.9 – 6.9 | 2.3 – 8.4 | 446 | 5.9 | 0.7 | 2.8 – 8.2 | 225 | 5.8 | 0.6 | 3.9 – 7.4 |
| Tapping – basic rate, part 3 (Hz) | 615 | 6.1 | 0.7 | 5.1 – 7.3 | 0.9 – 8.4 | 445 | 6.1 | 0.7 | 0.9 – 8.4 | 225 | 6.0 | 0.6 | 4.2 – 7.5 |
| Psychomotor endurance [Hz] | 615 | 5.9 | 0.7 | 4.8 – 7.0 | 0.1 – 8.2 | 445 | 5.9 | 0.7 | 0.1 – 8.2 | 225 | 5.8 | 0.6 | 3.4 – 7.5 |
| Viseomotor coordination ability (mistakes) [n] | 613 | 13.3 | 7.0 | 3.0 – 26.0 | 1.0 – 39.0 | 441 | 13.0 | 7.0 | 1.0 – 39.0 | 225 | 12.5 | 6.7 | 1.0 – 31.0 |
| Viseomotor coordination ability (time) [sec] | 613 | 29.7 | 14.2 | 12.8 – 57.8 | 6.5 – 103.2 | 441 | 30.8 | 14.4 | 6.6 – 103.2 | 225 | 32.6 | 14.5 | 11.2 – 103.2 |
| **Cognitive and mental parameters** | | | | | | | | | | | | | |
| Optical reaction time [msec] | 621 | 277.0 | 37.5 | 221.0 – 339.0 | 195 – 460 | 445 | 276.2 | 35.8 | 195 - 412 | 225 | 281.1 | 39.6 | 198 - 411 |
| Acoustical reaction time [msec] | 622 | 283.4 | 41.1 | 217.0 – 347.8 | 176 – 475 | 446 | 281.9 | 40.8 | 176 - 475 | 226 | 281.6 | 44.8 | 178 - 420 |
| Pursuing reaction time [msec] | 623 | 68.3 | 27.7 | 31.0 – 118.0 | 19 – 210 | 447 | 67.2 | 27.2 | 19 - 210 | 227 | 77.8 | 29.0 | 21 - 210 |
| Verbal reaction time [sec] | 619 | 10.8 | 1.8 | 8.2 – 14.0 | 7.2 – 19.9 | 447 | 10.6 | 1.8 | 7.2 – 19.9 | 227 | 11.2 | 1.9 | 7.6 – 19.9 |
| Cognitive reaction time [sec] | 619 | 12.9 | 2.3 | 9.8 – 16.8 | 7.9 – 24.0 | 447 | 12.8 | 2.3 | 8.4 – 24.0 | 227 | 13.5 | 2.5 | 8.4 – 24.0 |
| Cognitive switching capability [sec] | 619 | 22.7 | 7.7 | 14.6 – 34.8 | 10.4 – 113.0 | 447 | 22.8 | 8.1 | 10.4 – 113.0 | 227 | 25.9 | 9.3 | 13.0 – 113.0 |
| Ability to concentrate (mistakes) [n] | 618 | 1.5 | 2.0 | 0.0 – 6.0 | 0 – 14 | 446 | 1.5 | 3.0 | 0 - 14 | 227 | 2.0 | 2.2 | 0 - 11 |
| Ability to concentrate (time) [sec] | 618 | 121.2 | 37.8 | 73.0 – 189.0 | 52.1 – 320.4 | 446 | 122.3 | 38.0 | 52.1 – 320.4 | 227 | 132.5 | 40.4 | 57.1 – 320.4 |
| Orientation capability [n] | 621 | 50.0 | 14.2 | 38.0 – 71.0 | 30.0 – 181.0 | 447 | 50.6 | 15.2 | 30.0 – 181.0 | 227 | 53.6 | 18.7 | 30.0 – 181.0 |
| Strategic thinking [sec] | 621 | 152.6 | 67.2 | 86.8 – 260.6 | 64.3 – 779.9 | 447 | 153.3 | 71.6 | 66.0 – 779.9 | 227 | 178.3 | 87.0 | 67.3 – 779.9 |
| Memory performance [n] | 621 | 95.1 | 21.0 | 85.0 – 124.9 | 30 – 313 | 447 | 95.9 | 21.8 | 30 -313 | 227 | 100.1 | 28.5 | 30 – 313 |
| Change over capability [sec] | 621 | 1.04 | 0.40 | 0.62 – 1.61 | 0.5 – 6.6 | 447 | 1.04 | 0.43 | 0.5 – 6.6 | 227 | 1.16 | 0.53 | 0.5 – 6.6 |
| **Emotional-social parameters** | | | | | | | | | | | | | |
| Stress disposition [score] | 471 | 27.3 | 5.3 | 18.0 – 35.4 | 10.0 – 40.0 | 447 | 27.2 | 5.3 | 10.0 – 40.0 | 227 | 27.8 | 5.3 | 10.0 – 40.0 |
| Social dominance [score] | 471 | 26.8 | 5.2 | 17.0 – 35.0 | 9.0 – 40.0 | 447 | 26.6 | 5.2 | 9.0 – 40.0 | 227 | 26.8 | 5.5 | 9.0 – 40.0 |
| Social power [score] | 471 | 16.2 | 4.8 | 9.0 – 25.0 | 6.0 – 35.0 | 447 | 16.1 | 4.8 | 6.0 – 35.0 | 227 | 16.3 | 5.3 | 6.0 – 35.0 |
| Stress exposition [score] | 471 | 30.8 | 5.2 | 22.0 – 38.4 | 12.0 – 42.0 | 447 | 30.8 | 5.2 | 12.0 – 42.0 | 227 | 31.4 | 5.6 | 12.0 – 42.0 |
| Physical wellbeing [score] | 622 | 3.4 | 3.9 | 0.0 – 11.0 | 0.0 – 24.0 | 447 | 3.3 | 4.0 | 0.0 – 24.0 | 227 | 4.1 | 4.6 | 0.0 – 24.0 |
| Emotional wellbeing [score] | 622 | 1.7 | 2.6 | 0.0 – 7.8 | 0.0 – 14.0 | 447 | 1.8 | 2.7 | 0.0 – 14.0 | 227 | 1.8 | 2.5 | 0.0 – 14.0 |
| Complaint questionnaire - physical and emotional wellbeing [score] | 622 | 5.1 | 5.5 | 0.0 – 17.0 | 0.0 – 32.0 | 447 | 5.1 | 5.8 | 0.0 – 32.0 | 227 | 5.9 | 6.2 | 0.0 – 32.0 |
| Social activity / leisure [score] | 622 | 52.6 | 13.7 | 31.3 – 74.5 | 12.0 – 86.0 | 447 | 52.7 | 13.8 | 12.0 – 86.0 | 227 | 53.5 | 13.5 | 12.0 – 83.3 |
| Social activity / duties [score] | 622 | 90.3 | 11.0 | 70.5 – 106.5 | 50.0 – 118.5 | 447 | 90.1 | 11.3 | 50.0 – 118.5 | 227 | 93.6 | 10.6 | 50.0 – 118.5 |
| Sense of coherence [score] | 352 | 51.0 | 7.1 | 37.0 – 61.0 | 29.0 – 63.0 | 334 | 51.0 | 7.2 | 29.0 – 63.0 | 226 | 51.2 | 7.6 | 29.0 – 63.0 |
| Chronological age [years] | 625 | 39.6 | 14.8 | 22.3 – 62.9 | 20.0 – 78.7 | 447 | 39.6 | 14.8 | 20.2 – 78.7 | 227 | 52.3 | 8.2 | 35.1 – 69.2 |
| Bio-functional age (BFA) [years] | 321 | 44.0 | 8.7 | 29.3 – 57.6 | 20.1 – 73.7 | 227 | 43.9 | 8.7 | 20.1 – 73.7 | 227 | 43.9 | 8.7 | 20.1 – 73.7 |
| difference_Age_BFA_Index  (chronological – bio-functional age) | 321 | 8.3 | 8.1 | -5.4 – 21.0 | -19.4 – 30.6 | 227 | 8.6 | 8.3 | -19.4 – 30.6 | 227 | 8.6 | 8.3 | -19.4 – 30.6 |

Abbreviations: BFS: bio functional status, BFA: bio functional age, BFB: Biofeedback, DMF: Decayed Missinf filled teeth, N: Number, SD: standard deviation, Min: Minimum, Max: Maximum, BIA Bioelectrical impedance analysis, Xc Reactance (capacitive resistance), Rc Resistance (ohmic resistance), mmHG: Millimeter mercury column, Δ: delta, sec: seconds, msec: milliseconds, kg: kilograms, %: percentage, cm: centimeters, m^2^: square meter, dB Dezibel, Hz: Hertz, p0: reference pulse, n/min number per minute, l: Liter, KP: Kilopond, HV: hearing
