# Supplementary material for: Measuring Quality of Life: Incorporating Objectively Measurable Parameters within the Cross-Sectional Bern Cohort Study 2014 (BeCS-14)
Source: Int J Environ Res Public Health. 2024 Jan 15;21(1):94. doi: 10.3390/ijerph21010094 (PMC10815394; doi:10.3390/ijerph21010094)
Supplement: Supplementary file 1 [file ijerph-21-00094-s001.zip › Table S4.docx]

**Table_S_4:** Comparison of Total Cohort and Subgroups 1 and 2 (categorial values) and comparison of subgroup 1 who are not in subgroup 2

|  | **Total Cohort**  **(n=630)** | | **Subgroup 1**  **(n=447)** | **Subgroup 2**  **(n=227)** | **Subgroup 1**  **but not Subgroup 2**  **(n=220)** | **p**  **(subgroup 2**  **vs.**  **Subgroup 1-2)** |
| --- | --- | --- | --- | --- | --- | --- |
| **Parameter** | **%** | **95% CI** | **%** | **%** | **%** |  |
| Male  Female | 26.3  73.7 | 22.7 – 29.9  70.1 – 77.3 | 27.3  72.7 | 20.7  79.3 | 34.1  65.9 | 0.002 |
| Single, living alone, widowed, divorced or other | 25.3 | 21.8 – 28.9 | 23.7 | 21.2 | 26.4 | 0.221 |
| Living in a marriage | 30.6 | 26.9 – 34.4 | 31.3 | 56.4 | 5.5 | < 0.001 |
| Childless | 64.9 | 61.0 – 68.7 | 65.3 | 35.2 | 96.4 | < 0.001 |
| University degree | 39.9 | 30.2 – 37.9 | 38.1 | 29.5 | 46.8 | < 0.001 |
| Advanced technical college | 14.5 | 11.7 – 17.5 | 15.3 | 20.5 | 10.0 | 0.004 |
| Vocational training | 20.3 | 17.2 – 23.7 | 19.6 | 26.8 | 12.3 | < 0.001 |
| Monthly gross income  < 5000 CHF | 48.9 | 44.9 – 52.9 | 49.9 | 42.3 | 58.0 | 0.001 |
| Monthly gross income  > 5000 CHF | 41.1 | 37.2 – 45.1 | 42.3 | 55.0 | 28.8 | < 0.001 |
| No own income | 8.7 | 6.6 – 11.3 | 7.4 | 1.8 | 13.2 | < 0.001 |
| Professional field:  Social  Economy  Jobless | 37.3  18.7  3.0 | 33.5 – 41.3  15.7 – 22.1  1.8 – 4.7 | 36.7  17.4  3.4 | 49.8  19.8  3.5 | 23.3  15.0  3.2 | 1.000  0.283  1.000 |
| Employee status:  Executive position  Employed  Student | 17.1  51.7  23.5 | 14.2 – 20.3  47.7 – 55.8  20.2 – 27.1 | 17.0  52.1  23.3 | 26.0  67.4  0.4 | 7.8  36.5  47.0 | < 0.001  < 0.001  < 0.001 |
| Job occupation  > 90%  50-89%  < 50% | 41.6  26.7  21.7 | 37.7 – 45.6  23.2 – 30.4  18.5 – 25.2 | 43.4  26.8  21.9 | 39.2  38.8  16.7 | 47.9  14.8  27.4 | 0.070  < 0.001  0.008 |
| Non smoking | 65.0 | 61.2 – 68.9 | 65.5 | 61.2 | 70.0 | 0.059 |
| Physical activity till sweating  1-2 times/week  > 2 times/week | 37.3  37.3 | 33.5 – 41.3  33.5 – 41.3 | 36.7  38.0 | 41.0  28.2 | 32.3  48.2 | 0.062  < 0.001 |
| Alcohol consumption  up to 4 times / month | 56.2 | 52.2 – 60.2 | 56.8 | 53.1 | 60.5 | 0.152 |
| Alcohol consumption  up to 4 times / week | 6.2 | 4.4 – 8.4 | 6.7 | 7.5 | 5.9 | 0.573 |
| Daily alcohol consumption  up to 3 glasses | 35.2 | 31.5 – 39.2 | 34.9 | 37.0 | 32.7 | 0.372 |
| Disease – free | 24.1 | 20.8 – 27.7 | 24.6 | 25.1 | 24.1 | 0.827 |
| No Medication use | 42.9 | 38.9 – 46.9 | 42.7 | 33.1 | 52.7 | < 0.001 |

Abbreviations: CI: Confidence interval of percentage, n: number, %: percentage, CHF: swiss franks.

**Table 1. b** Comparison of Total Cohort and Subgroups (metric values).

|  | **Total Cohort**  **(n=630)** | | | | **Subgroup 1**  **(n=447)** | | | **Subgroup 2**  **(n=227)** | | |
| --- | --- | --- | --- | --- | --- | --- | --- | --- | --- | --- |
| **Parameter** | **Mean**  **(SD)**  **%** | **95% CI** | **Median**  **Q1, Q3** | **Mean**  **(SD)**  **%** | | **Median**  **Q1, Q3** | **Mean**  **(SD)**  **%** | | **Median**  **Q1, Q3** |  |
|  |  |  |  |  | |  |  | |  |  |

| Age | 39.5  (14.9) | 38.3 – 40.7 | 37.0  25.0, 53.0 | 39.6  (14.9) | 36.0  25.0, 53.0 | 52.4  (8.1) | 53.0  47.0, 59.0 |
| --- | --- | --- | --- | --- | --- | --- | --- |

|  |  |  |  | 26.4  (6.1) | 25.0  23.0,59.0 | < 0.001 | |
| --- | --- | --- | --- | --- | --- | --- | --- |
| Children | 1.76  (1.17) | 1.6 – 1.9 | 1.0  1.0, 3.0 | 1.8  (1.2) | 1.0  1.0, 3.0 | 2.5  (1.3) | 3.0  1.0, 3.0 |

|  |  |  |  | 1.1  (0.5) | 1.0  1.0,1.0 | < 0.001 | |
| --- | --- | --- | --- | --- | --- | --- | --- |
| Smoking (cigarettes/day) | 1.62  (1.06) | 1.53 - 1.7 | 1.0  1.0, 2.0 | 1.60  (1.01) | 1.0  1.0, 2.0 | 1.58  (0.91) | 1.0  1.0, 2.0 |
|  |  |  |  | 1.60  (1.17) | 1.0  1.0,2.0 | 0.251 | |

Abbreviations: CI: Confidence interval of percentage, n: number, %: percentage, SD: standard deviation, Q1: Quartile 25%, Q3 Quartile 75%.
